# The α_1_-adrenergic receptors: diversity of signaling networks and regulation

**DOI:** 10.3109/10799893.2010.518152

**Published:** 2010-10-18

**Authors:** Susanna Cotecchia

**Affiliations:** Dipartimento di Fisiologia Generale e Ambientale, Università di Bari, Italy, and Département de Pharmacologie etde Toxicologie, Université de Lausanne, Switzerland

**Keywords:** Constitutive activity, oligomerization, β-arrestin, protein interactions, knock outmice, transgenic mice

## Abstract

The α_1_-adrenergic receptor (AR) subtypes (α_1a_, α_1b_, and α_1d_) mediate several physiological effects of epinephrineand norepinephrine. Despite several studies in recombinant systems and insightfrom genetically modified mice, our understanding of the physiological relevance and specificity of the α_1_-AR subtypes is still limited. Constitutive activity and receptor oligomerization have emerged as potential features regulating receptor function. Another recent paradigm is that βarrestins and G protein-coupled receptors themselves can act as scaffolds binding a variety of proteins and this can result in growing complexity of the receptor-mediated cellular effects. The aim of this review is to summarize our current knowledge on some recently identified functional paradigms and signaling networks that might help to elucidate the functional diversity of the α_1_-AR subtypes in various organs.

## Introduction

Within the large family of G protein-coupled receptors (GPCR), the adrenergic receptors (ARs) mediate the functional effects of catecholamines, like epinephrine and norepinephrine. The AR family includes nine different gene products, three β (β_1_, β_2_, β_3_), three α_1_ (α_1a_, α_1b_, and α_1d_), and three α_2_ (α_2A_, α_2B_, and α_2C_) receptor subtypes.

The α_1_-AR subtypes are expressed in various organs, including brain, heart, blood vessels, liver, kidney, prostate, and spleen, in which they mediate a variety of functional effects such as modulation of neurotransmission, vasoconstriction, cardiac inotropy, and chronotropy, regulation of metabolism (reviewed in ref. 1). Activation of the three α_1_-AR subtypes causes polyphosphoinositide hydrolysis catalyzed by phospholipase C (PLC) via pertussis toxin-insensitive G proteins in most tissues where this effect has been examined (1).

Radioligand binding studies in rat tissues initially demonstrated two classes of α_1_-AR binding sites, “A” and “B” with high and low affinity for the α_1_-AR antagonists WB4101 and phentolamine, respectively. The first α_1_-AR cloned, was unequivocally assigned to the pharmacological α_1B_ subtype and hence named α_1b_-AR. The pharmacological α_1A_ subtype, today identified as α_1a_-AR, was initially cloned from a bovine brain library and inappropriately named α_1c_-AR or α_1A/C_-AR. Finally, the cloned α_1d_-AR (initially named α_1A_-AR or α_1A/D_-AR) was a novel receptor subtype not clearly identified by previous pharmacological studies (reviewed in ref. 2,3).

Studies aiming to assess the specific functional responses mediated by distinct α_1_-AR subtypes have been hampered by the fact that the subtype-selective drugs are only moderately selective. Recently, studies on genetically modified mice lacking or overexpressing one or more α_1_-AR subtypes have provided some important insight into the functional roles played by distinct receptors. However, our understanding on the functional implications of α_1_-AR heterogeneity in physiological systems is still quite limited.

Extensive mutational analysis performed by our group and other investigators helped to identify the structural determinants of the α_1_-AR subtypes involved in each of the three main “classical” functional properties of GPCRs: (i) ligand-binding; (ii) receptor activation and coupling to G protein; and (iii) desensitization. These findings have been reviewed elsewhere (4,5). Beyond these “classical” features, a number of novel functional paradigms have been recently described for GPCRs including receptor constitutive activity (6), oligomerization (7) and interaction with a variety of signaling proteins (8). These functional features imply a growing complexity of signaling and regulation of the α_1_-AR subtypes which might represent the mechanistic basis of their functional specificity in various tissues.

The aim of this review is to summarize our current knowledge on some recently identified functional paradigms and signaling networks that might help to elucidate the functional diversity of the α_1_-AR subtypes in various organs.

## Constitutive activity of the α_1_-AR subtypes

For both the α_1a_ and α_1b_-AR mutation-induced and spontaneous constitutive activity have been reported (9,10). Interestingly, most of the known α-blockers behave as inverse agonists both at the wild type and constitutively actve mutants of the two receptors (10). Studies on constitutively activating mutations of the α_1b_-AR provided important insight into the potential molecular mechanisms of GPCR activation (11). In particular, they highlighted the highly conserved E/DRY sequence at the cytoslic end of helix 3 as an important switch of receptor activation.

Interestingly, activating mutations which perturb the helix 3/helix 6 packing of the receptor have been found in both the α_1a_ and α_1b_-AR subtypes suggesting common mechanisms of receptor activation (12). These include: (i) mutations of A293^(6.34)^ and of A271^(6.34)^ in the cytosolic extension of helix 6 in the α_1b_-AR and α_1a_-AR, respectively (9,10); (ii) mutations of D142^(3.49)^ and D123^(3.49)^ of the E/DRY motif in the α_1b_-AR and α_1a_-AR, respectively (10,11).

However, some differences in the activation properties can be observed between the α_1a_ and α_1b_-AR in recombinant systems measuring the inositol phosphate response. The agonist-independent activity of both the wild type α_1b_-AR and its constitutively active mutants was significantly higher than that of the wild type α_1a_-AR or its mutant. In contrast, the epinephrine-induced inositol phosphate accumulation above basal at the α_1a_-AR was significantly higher than that at the α_1b_-AR or its constitutively active mutants expressed at comparable levels (10,13). This suggests that in recombinant systems the agonist-occupied α_1a_-AR has greater efficacy in activating PLC than the α_1b_-AR whereas its spontaneous or mutation-induced isomerization toward the active states is lower. Only one study reported the opposite showing that in rat neonatal cardiomyocytes a different constitutively active mutant of the α_1a_-AR displayed higher basal activity than the analogous mutant of the α_1b_-AR (14). This finding is intriguing and should be further explored.

The properties of the α_1d_-AR subtype have been investigated very little because its expression as well as the agonist-induced inositol phosphate response mediated by this receptor were often found to be much smaller than those of the other two subtypes (15,16). Constitutively activating mutations of the α_1d_-AR have not been reported so far. However, an interesting study reported that the α_1d_-AR expressed in rat fibroblasts is constitutively active and internalized (17). In fact, the basal activity of the α_1d_-AR was 2-fold greater than that of the α_1b_-AR and was increased following the treatment with the inverse agonist prazosin which caused its redistribution from the intracellular compartments to the plasma membrane. The constitutive activity of the α_1d_-AR was also observed in physiological systems like in aorta and mesenteric arteries where it could inhibited by inverse agonists (18). For the α_1a_ or α_1b_-AR constitutive activity in physiological systems has not been investigated.

Altogether, these findings indicate that there might be important differences in the constitutive activity of the α_1_-AR subtypes which could have consequences in their signaling and regulatory properties *in vivo*. Such differences should be further explored and the elucidation of their physiological implications might represent an important area of investigation.

## Oligomerization of the α_1_-AR subtypes

Findings in the last decade challenged the widely held view of GPCRs functioning as monomeric units. Co-immunoprecipitation of differentially tagged GPCRs or functional complementation of pairs of co-expressed inactive receptor mutants provided strong evidence that GPCR oligomers do exist. The widespread use of biophysical techniques such as fluorescence resonance energy transfer (FRET) or bioluminescence resonance energy transfer (BRET) between GPCRs carrying the appropriate pair of fluorescent/bioluminescent labels suggested oligomerization of a variety of GPCRs. Each technique employed has its own shortcomings: whereas co-immunoprecipitation cannot rule out indirect interaction, energy transfer techniques can only certify that the two partners are in close proximity, not necessarily in immediate contact. However, convergent results obtained through independent methods eventually led to the widespread acknowledgment of the existence of GPCR oligomers (7).

Both homo- and hetero-oligomerization have been demonstrated for the three α_1_-AR subtypes in recom-binant systems (Table 1) (15,16,19). FRET measurements as well as co-immunoprecipitation experiments provided evidence that both the α1a and α1b-AR can form homo-oligomers (19). Oligomerization of the α_1b_-AR did not require the integrity of its C-tail, of two glycophorin motifs or of the N-linked glycosylation sites at its N-terminus. Constitutively active or non-functional α_1b_-AR mutants displayed the same propensity to oligomerize as the wild-type receptor, indicating that the activation state of the receptor is irrelevant for this process. Receptor oligomerization was not influenced by the agonist epinephrine or by the inverse agonist prazosin. Thus, whether homo-oligomerization of the α_1a_ or α_1b_-AR has any functional relevance is unknown.

**Table 1:** Oligomerization of the α_1_-adrenergic receptor subtypes.

Receptors	Trafficking	Pharmacology	Signaling	Ref.
α_1a_/α_1b_	Co-endocytosis	No change	–	19
α_1b_/α_1d_	↑ α_1d_ Surface expression	↓ α_1d_ Affinity for selective ligands	↑ Signaling	15
α_1d_/β_2_	↑ α_1d_ Surface expression co-endocytosis	–	–	16
Homooligomers α_1a_, α_1b_, α_1d_	–	–	–	15,19

Hetero-oligomers were observed between the α_1a_ and the α_1b_-AR subtypes, but not between the α_1b_-AR and other GPCRs. Interestingly, hetero-oligomerization was found to have an impact on receptor endocytosis (19). Whereas the α_1b_-AR undergoes agonist-induced inter-nalization, the α_1a_-AR does not. However, when the two AR subtypes were co-expressed forming heterodimers, the endocytosis of each monomer could be triggered by stimulation of the other. Colocalization of the two monomers could be seen in endocytic vesicles suggesting that the α_1a_/α_1b_ dimers remained stable throughout the endocytosis process.

An important effect of hetero-oligomerization has been reported for the α_1d_-AR subytpe. In fact, co-expression of the α_1d_AR with the α_1b_AR (15) or the β_2_AR (19) was able to rescue surface expression of the α_1d_-AR, the majority of which is intracellular when expressed alone in various cell lines. Interestingly, the interaction with the α_1b_-AR modified the pharmacological profile of the α_1d_-AR which looses its affinity for its selective ligand BMY7378 when it is co-expressed with the α_1b_-AR. The α_1b_/α_ld_ dimer behaves as a single functional entity with increased response to norepinephrine relative to either monomer alone. The α_1d_-AR receptor was long supposed to be little expressed in the heart, as its selective ligand BMY7378 could detect only minimal levels of the receptor. However, these findings should be considered in a new light, given that the α_1b_ and α_1d_-AR subtypes co-exist in various tissues and the pharmacological profile of the α_1d_-AR might be different than expected because of oligomerization.

Oligomerization of α_1_-AR subytpes in physiological systems has not been explored so far for lack of appropriate experimental tools. Therefore, the functional relevance of α_1_-AR oligomerization *in vivo* remains elusive. However, oligomerization might represent an additional mechanism regulating the physiological responses mediated by the α_1_-AR subytpes which are often co-expressed in the same cells. Further exploring the functional correlates of receptor oligomerization and assessing if it occurs in physiological systems might provide interesting information about cross-talk effects at the level of α_1_-AR signaling or regulation.

## Signaling pathways of the α_1_-AR subytpes

It has become increasingly evident that the variety of functional effects mediated by the α_1_-ARs in different organs must imply the activation of multiple signaling pathways beyond activation of PLC via Gq/11. Therefore, several studies have attempted to investigate whether each α_1_-AR subtype may activate distinct signaling pathways, but our knowledge on this issue is still limited.

It has been reported that stimulation of the α_1b_ and α_1d_-AR can result in the activation of phospholipase A2 in COS-1 cells (20); the α_1a_-AR was not explored. In NIH3T3 cells, the activation of the α_1a_ and α_1b_-AR, but not that of the α_1d_, resulted in the stimulation of p21-ras, PI3-kinase and mitogen-activated protein kinase (MAPK) (21). However, the steps leading to the activation of these pathways seem to differ between the two receptor subtypes.

In hepatocyte derived cells, stimulation of the α_1b_-AR subtype inhibits interleukin 6 signaling by a MAPK mechanism (22). An interesting microarray study indicated that the α_1_-AR subtypes expressed in Rat fibroblasts have a differential effect on cell cycle genes with the α_1b_ mediating cell-cycle progression, and the α_1a_ and α_1d_-AR mediating G1-S cell cycle arrest (23).

Most of the work investigating α_1_-AR signaling has been performed in cardiomyocytes. In fact, hearts of most species express both α_1a_ and α_1b_-AR at protein level whereas the expression of α_1d_-AR is very low. The α_1a_-AR predominates in humans, whereas the α_1b_-AR in rodents. Some seminal studies (24,25) demonstrated that stimulation of the α_1_-ARs in cardiomyocytes induces a hypertrophic response accompanied by the activation of early genes (c-fos, c-jun, egr-1) upreagulation of contractile proteins (myosin light chain-2) and reactivation of embryonic genes (atrial natriuretic factor (ANF), β-myosin heavy chain, skeletal α-actin).

Various studies provided clear evidence for the involvement of both the PLC–MAPK pathway (26) and Rho-signaling (27) in the α_1_-AR-induced hypertrophic response in cardiomyocytes. A recent study supports these earlier findings suggesting that α_1_-AR-induced cardiac hypertrophy is mediated by three parallel pathways: G12/13-Rho-JNK, Gq-JNK (Rho-independent) and Gβγ (JNK independent) (28). Recent findings have demonstrated that the α_1_-ARs endogenously expressed in rat neonatal cardiomocytes promote RhoA-activation via a mechanism that requires G12 and the Rho-guanine nucleotide exchange factor AKAP-Lbc and this pathway mediates hypertrophy (29).

The respective role in stimulating cardiac hypertrophy of the two α_1_-AR subtypes expressed in heart, the α_1a_ and α_1b_-AR, does not emerge clearly from the *in vitro* studies published so far probably because of the limited selectivity of the pharmacological tools available. In one study on rat neonatal cardiomyocytes, a constitutively active form of the α_1a_-AR activated gene expression of the ANF, whereas the analogous constitutively active mutant of the α_1b_-AR stimulated gene expression of c-fos, but not of ANF (14). However, these findings are intriguing considering that other studies reported the opposite and that overexpression of the α_1b_-AR in transgenic mice resulted in a marked increase in ANF (see below). In the future, it would be interesting to carry on a systematic investigation of different signaling pathways comparing the α_1_-AR subtypes expressed in the same cellular systems and to correlate these findings with the growing information provided by *in vivo* studies on genetically modified mice (see below).

## Regulatory mechanisms and Parrestin interaction at the α_1_-AR subytpes

The α_1_-AR subtypes display quite divergent regulatory properties. In fact, the α_1b_-AR in recombinant systems undergoes rapid phospohorylation, desensitization and endocytosis upon exposure to the agonist (30–32). Desensitization involves phosphorylation of residues in the C-tail of the receptor mediated by G protein-coupled receptor kinases (GRKs) (31). The endocytosis of the α_1b_-AR occurs via clathrin-coated vesicles and seems to involve βarrestins (32).

In contrast, the α_1a_-AR expressed in rat-1 fibroblasts is poorly phosphorylated and desensitized compared to the α_1b_-AR (33). In addition, it undergoes very modest agonist-induced endocytosis (32).

Fewer studies have investigated the desensitization of the α_1d_-AR probably because of its poor expression in recombinant systems. It has been reported that noradrenaline and direct activation of protein kinase C induce phosphorylation of the α_1d_-AR and this correlates with desensitization of the receptor (34). However, desensitization of the α_1d_-AR was not compared with that of the other two subtypes in this study.

Overall, the impact of α_1_-AR desensitization in physiological systems where the receptors are endogenously expressed has been poorly investigated, as it is the case for most GPCRs. Therefore, what is the impact of different regulatory properties of the α_1_-AR subtypes on complex functions like vasoconstriction, metabolic response, and others, is unknown.

Interestingly, the different regulatory features of the α_1a_ and α_1b_-AR seem to correlate with their pattern of interaction with βarrestins. In fact, the results from both co-immunoprecipitation experiments and βarrestin translocation assays indicated that the agonist-induced interaction of the α_1a_-AR with βarrestin was much weaker than that of the α_1b_-AR (32). The interaction of βarrestin with the α_1d_-AR has not been directly explored so far.

These differences in receptor/βarrestin interaction might have broader implications in α_1_-AR mediated signaling because of the well established role played by βarrestins in coordinating a variety of signaling networks (35). In particular, it is well established that βarrestins are scaffolds for components of the MAPK cascade thus mediating MAPK activation induced by various GPCRs. Investigation of βarrestin-mediated signaling at the α_1_-AR subtypes is an interesting area of investigation which has been poorly explored so far and might represent one of the mechanisms contributing to the variety of the receptor-mediated-responses.

## Proteins interacting with different α_1_-AR subtypes

One of the most recent paradigms is that GPCRs can bind a variety of proteins and this can promote multiple signaling events which results in growing complexity of the receptor-mediated cellular effects (8)

A number of approaches have been followed to identify novel proteins interacting with the α_1_-ARs, including yeast two-hybrid screen using cytosolic portions of the receptors as bait, pull-down or *in vitro* overlay assays using purified proteins, co-immunoprecipitation of receptor-protein complexes from recombinant or native cells, FRET or BRET technology in cells. These studies resulted in the identification of a variety of proteins interacting with the α_1_-AR subtypes, several of them in a receptor subtype selective pattern (Table 2).

**Table 2:** Proteins interacting with the α_1_-adrenergic receptor subtypes.

Receptor	Partner	Binding site	Functional role	Ref.
α_1a_ α_1b_ α_1d_	nNOS	Unknown	Unknown	36
α_1a_	Tolloid	C-tail	↓ Surface expression	37
α_1a_	RGS2	i3 loop(K219-S220-R238)	↓ Gq signaling	38
α_1b_	AP50	C-tail (8 Arg)	↑ Endocytosis	40
α_1b_	Ezrin	C-tail (8 Arg)	↑ Recycling	41
α_1b_	Spinophilin	i3 loop	↓ Ca^2+^ signaling induced by RGS2	43
α_1d_	Syntrophins	C-term (ETDI)	Stabilization of receptor at cell surface	44
α_1b_ α_1d_	gC1qR	C-tail (Arg)	Unknown	42

The α_1a_-AR subtype contains a PDZ binding sequence G-E-E-V at its C-terminus that can be expected to give rise to PDZ-domain mediated interactions. An early report, at the issue of a yeast two-hybrid screen, identified the type III PDZ domain of nNOS (neuronal nitric oxide synthase) as a potential α_1a_-AR interacting protein (36) However, co-immunoprecipitation studies, while confirming this interaction, failed to highlight selectivity for the α_1a_-AR subtype since all three α_1_-AR subtypes could be co-im-munoprecipitated with nNOS and this even when they were lacking their C-terminus. This interaction appeared to be without apparent physiological implications in spite of the known role of NO in the regulation of blood pressure and of nNOS as local metabolic inhibitor of α_1_-AR-mediated vasoconstriction.

Another study reported that the CUB5 domain of mammalian tolloid (mTLD), a zinc-finger matrix metal-loprotease of the astacin family, interacted with α_1a_-AR C-tail in a yeast two hybrid screen (37). Overexpression of mTLD reduced the number of cell surface receptors without affecting total receptor level or affinity when transiently expressed in HEK293 cells. No mechanism was proposed to account for the observed phenomena.

Interesting prospects were opened by the report of the direct interaction between RGS2 (regulator of G protein signaling 2) and the third intracellular loop of the α_1a_-AR (38). RGS proteins are well characterized inhibitors of heterotrimeric G protein function, acting as GAPs (GTPase activating proteins) to increase the rate of GTP hydrolysis at Gα subunits and thus terminate signaling. More than 30 RGS proteins have been identified so far, but many RGS proteins can non-selectively bind to and inhibit Gαi/o and Gαq11 in reconstituted systems, suggesting that other factors may regulate their specificity for a particular signaling pathway. RGS2 was found to interact with the α_la_-AR third intracellular loop confirming what previously shown for other Gq-coupled receptors, namely the cholinergic mus-carinic M1, M3, and M5 receptors (39) and it inhibited agonist-induced inositol phosphate responses without affecting ligand binding.

Two main interacting partners were pulled out of a yeast two-hybrid screen for the α_1b_-AR: the μ2 (or AP50) subunit of the clathrin adaptor complex AP2 (40) and ezrin, a member of the ezrin-radixin-moesin (ERM) protein family (41). The AP2 complex is part of the endocytic machinery mediating clathrin-dependent endocytosis of membrane proteins and it is recruited to agonist-activated GPCRs through the intermission of βarrestins. Binding of AP50 relied on a basic stretch of eight arginines in the proximal C-tail of the receptor. Direct association of the α_1b_-AR to AP50 contributed to the agonist-induced internalization of the receptor as demonstrated by the fact that a receptor mutant lacking the AP50 binding motif was delayed in internalization. The presence of the eight arginine motif in the C-tail of a GPCR is not common, which rules out the hypothesis that direct AP50 interaction is a common mechanism for clathrin-mediated endocytosis. Interestingly, this feature is shared by the α_1d_-AR, which contains a stretch of seven positive charges in its C-tail, but no studies were undertaken using this receptor subytpe.

In addition to AP50, the same yeast two-hybrid screen identified ezrin as a potentially direct binding partner of the α_1b_-AR (41). Ezrin belongs to the ERM family of proteins, primarily described as linkers between membrane proteins and cortical actin. Ezrin interactions with polytopic membrane proteins generally occur through the adaptor proteins EBP50 (NHERF1) and E3KARP (NHERF2). So far, a role for the ERM proteins in GPCR trafficking was inferred from the finding that NHERF1 binding to some GPCRs promoted their recycling, depending on its binding to ERM proteins. The α_1b_-AR is the first GPCR for which a direct interaction with ezrin has been found. Disruption of this interaction by overexpression of a dominant negative mutant of ezrin inhibited receptor reycling after internalization, as did actin depolymeri-zation. However, ezrin was also shown to be involved in the remodelling of the actin cytoskeleton, in the modulation of Rho-signaling (by binding to Rho-GTP dissociation inhibitor and thourgh direct association to several Rho-GTP/GDP exchange factors) as well as in anchoring of protein kinase A. Therefore, it would be interesting to test whether ezrin is also involved in ρ-signaling mediated by the α_1b_-AR.

Another protein, the receptor for globular “Heads” of c1q (gC1qR), was reported to interact with the same arginine-rich sequence in the α_1b_ and the α_1d_-AR (42). gC1qR is a glycoprotein mainly displaying intracellular localization, but also present on the surface of macro-phages and T cells through anchoring to β-integrin, where it is part of a complement receptor. No functional relevance was demonstrated for its interaction with the α_1_-ARs.

An interesting protein found to interact with the α_1b_-AR is spinophilin which interacts with other GPCRs, including the α_2_-AR, as well as with the N-terminal domain of RGS proteins (RGS1, 2, 4, and 16) which participates in GPCR recognition (43). Thus spinophilin might represent an interesting functional bridge between RGS and α_1_AR subtypes that don't bind RGS, like the α_1b_AR. In fact, it has been found that spinophilin increases the RGS2-induced inhibition of the α_1b_-AR calcium response. These data offer a glimpse into a potentially more general regulatory mechanisms of GPCR function by spinophilin.

The α_1d_-AR was for a long time a “poor relative” to the other α_1_AR subtypes, the α_1A_ and α_1B_ because poorly expressed at the cell surface in heterologous systems, probably because of its long N-terminus. This peculiarity hampered the investigation of its potential interactions with other proteins. Apart from the above mentioned interaction with gC1qR, whose functional implications are unknown (42), another interacting partner of the α_1d_-AR was a-syntrophin (44). α-syntrophin, a protein containing one PDZ domain and two PH (pleckstrin homology) domains, specifically recognized the C-tail of the α_1d_-AR, but not that of the α_1a_ or α_1b_, in the yeast two-hybrid assay. The PDZ domains of syntrophin isoforms α, β1, andβ2, but not γ1 or γ2, could interact with the α_1d_AR C-tail. The α_1d_-AR possesses the C-terminal sequence E-T-D-I, whose mutation impaired syntrophin binding to the receptor and markedly decreased norepinephrine-induced inositol phosphate accumulation. This mutation also dramatically decreased receptor expression levels. Taken altogether these results suggested that syntrophins act to maintain the stability of the α_1d_-AR through a PDZ-mediated interaction.

Altogether these findings indicate a rather complex and heterogeneous pattern of receptor/protein interactions whose physiological implications are far from being fully elucidated. The direct interaction of α_1_AR subtypes with selected partners identified in recombinant systems might result in new mechanisms of receptor signaling and regulation. Since these mechanisms might be specific for distinct receptors or cell types, the study of these interactions is an interesting approach to better understand the functional specificity of the receptors. However, this would require a systematic proteomic approach in different tissues expressing the α_1_AR subtypes as well as good experimental tools to investigate its functional implications.

## Insights from genetically modified mice

Recently, mouse lines carrying genetic modifications of the α_1_-AR subtypes have provided interesting information on the *in vivo* functions of the receptors giving some insight into the specificity of their role. The α_1b_-AR knock out (KO) mouse was the first model to be created (45) and it was characterized for a number of functional parameters. The α_1b_KO mice displayed: (i) decreased blood pressure response to phenylephrine with normal resting pressure (45); (ii) hyperinsulinemia, insulin resistance and high fat diet-induced obesity (46); and (iii) behavioral changes including blunted locomotor response to drugs of abuse and markedly decreased sensitivity to morphine and cocaine (47). Other mice carrying genetic modifications of the α_1_-AR subtypes have been mainly characterized for their cardiovascular phenotype (Table 3) thus allowing to build a more comprehensive picture of the functional role of each receptor in the cardiovascular system.

**Table 3:** Cardiovascular phenotype of mice carrying genetic modifications of different α_1_-adrenergic receptor subtypes.

Receptor	Genetic modification	Phenotype	Ref.
α_1b_	Gene deletion	↓ Resting blood pressure	48
		↓ Blood pressure response to phenylephrine	
α_1a_	Overexpression/heart-specific promoter	↑ Contractile response	54
		↑ Survival	
		↑ ANF mRNA	
		No hypertrophy	
		↑ Post-ischemic protection	
α_1b_	Gene deletion	Normal resting blood pressure	45
		↓ Blood pressure response to phenylephrine	
		↓ Vasoconstriction	
α_1b_	Overexpression/heart-specific promoter	↑ Phospholipase C activity	55
		↑ ANF mRNA	
		No hypertrophy	
		↓ Contractile and heart rate response to β-AR	
CAM α_1b_	Overexpression/heart-specific promoter	↑ Phospholipase C activity	50
		↑ Hypertrophy	
		↑ ANF mRNA	
		Normal blood pressure	
CAM α_1b_	Overexpression/receptor promoter	↓ Contractile response to β-AR	52
		Autonomic failure	
		↑ Hypertrophy	
α_1a_ α_1b_	Double gene deletion	*In males*Normal resting blood pressure	53
		↓ Cardiac growth after birth	
		↓ Heart rate, ↓ cardiac output	
		↓ Basal ERK activity	
		↑ Mortality to pressure overload	
		Contraction abnormalities	
α_1d_	Gene deletion	↓ Resting blood pressure	49
		↓ Blood pressure response to phenylephrine	
		↓ Vasoconstriction	
α_1d_ α_1b_	Double gene deletion	↓ Resting blood pressure	57
		↓↓ Blood pressure response to phenylephrine	
		↓↓ Vasoconstriction	

Both the α_1a_ and α_1d_-AR KO mice displayed decreased resting blood pressure as well as phenylephrine stimulated pressure response (48,49). The fact that the acute response to phenylephrine is decreased in all three KO mice indicates that the α_1a_, α_1b_ and α_1d_-AR all contribute to the regulation of the vascular tone. However, the contribution of the α_1a_ and α_1d_-AR subtypes is prominent because deletion of either one of the two receptors leads also to decreased resting blood pressure. This can be explained by the fact that the α_1a_-AR prevails in distributing arteries (mesenteric, renal) (48) and the α_1d_-AR in large conducting arteries (aorta and carotid) (49), whereas the expression of the α_1b_-AR is minor in all arteries.

Studies on genetically modified mice have also provided interesting insight into the role of the α_1_-AR in cardiac function and hypertrophy. As mentioned above, the α_1a_ and α_1b_-AR subtypes are both expressed in cardiomyo-cytes with the α_1a_ predominating in humans and the α_1b_ in rodents. Transgenic mice overexpressing a constitutively active α_1b_-AR mutant specifically in the heart display cardiac hypertrophy without any change in blood pressure (50). This supports previous evidence that stimulation of α_1_-ARs in cardiomyocytes *in vitro* leads to a hypertrophic response (24). This finding is also consistent with the role played by the Gq/PLC pathway in heart as demonstrated by the fact that transgenic mice overexpressing a constitutively active Gaq develop cardiac hypertrophy (51).

Interestingly, another transgenic mouse overxepress-ing a different constitutively active α_1b_-AR mutant, under the control of the receptor own promoter, displayed a more complex phenotype characterized by cardiac hypertrophy as well as autonomic failure (52). This confirms a direct role of the α_1b_-AR in cardiac hypertrophy, but indicates that broader effects occur when the receptor is generally overexpressed.

Mice overexpressing constitutively active mutant of the α_1a_-AR subtype have not been generated. However, the role of the α_1a_-AR in heart growth *in vivo* has been demonstrated by studies on double KO mice carrying deletions of both the α_1a_ and α_1b_-AR (53) which displayed several abnormalities including: (i) reduced growth of the heart after birth; (ii) reduced cardiac output; and (iii) increased mortality after pressure overload. These findings demonstrate that both the α_1a_ and α_1b_-AR play an important role in heart growth after birth and their integrity is required to maintain correct heart function.

These changes were, however, sex specific since they were observed in males, but not in females. This might be explained by the fact that females have a lower sympathetic tone and the growth of their hearts is less dependent on the α_1_-ARs.

Cardiac hypertrophy was not observed in transgenic mice with cardiac-specific overexpression of the wild type α_1a_ or α_1b_-AR subtype (54,55) despite the fact that they displayed increased expression of ANE This is unlike the phenotype of mice overexpressing the constitutively active α_1b_-AR mutant (50). This difference might be due to the fact that the signaling of a constitutively active mutant is somehow different or has greater efficacy than that of the wild-type receptor.

However, transgenic mice overexpressing either the α_1a_ or α_1b_-AR subtype in the heart provided a number of novel findings on the functional role of these receptors in heart. In fact, in the heart of the α_1b_-AR transgenic mice left ventricular contraction in response to β-agonists was depressed (55). Interestingly, it was found that dampening of β-AR signaling through adenylate cyclase was due to activation of a pertussis-sensitive inhibitory G protein. This clearly suggests that when overexpressed α_1_-ARs can couple to inhibitory G proteins.

**Figure 1:**
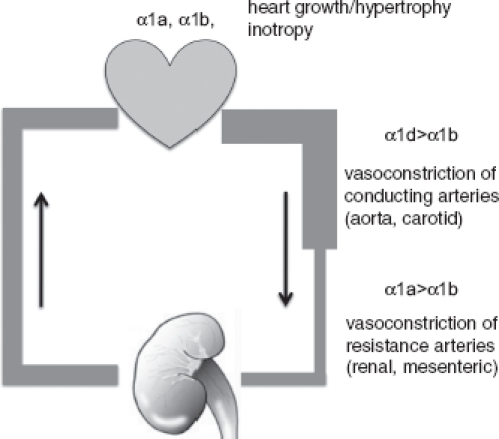
The α_1_-adrenergic receptor subtypes in the cardiovascular system. This figure summarizes the main roles played by distinct α_1_-AR subtypes in the cardiovascular system highlighted by studies on genetically modified mice.

In conclusion, as summarized in Figure 1, studies on mice carrying genetic modifications of the α_1_-AR genes have provided evidence that: (a) all three α_1_-AR subtypes contribute to the regulation of blood pressure with a prominent role for the α_1a_ and α_1d_; (b) both the α_1a_ and α_1b_-AR play a role in cardiac pathological hypertrophy (independent from pressure overload) or physiological hypertrophy associated with postnatal growth; and (c) the α_1_-ARs maintain normal heart function as demonstrated by the fact that the double deletion of the α_1a_ and α_1b_-AR results in some features of failing heart.

Other interesting features of the α_1_-AR subtypes have emerged from studies on the genetically modified mice including their effects on heart contractile function, cardiac rhythm and protection from ischemic injury (56). Additional studies are required to gain a deeper understanding of these complex effects.

## Conclusions and perspectives

In the past years, we have gained significant information of some molecular properties and functional implications of the α_1_-AR subtypes both from *in vitro* and *in vivo* studies.

Several studies focused on individual receptor subtypes whereas only few others attempted to compare the behavior of different receptors in similar experimental conditions. This latter approach should be implemented in future studies, both *in vitro* and *in vivo*, to better assess differences and similarities among the three α_1_-AR subtypes.

The elucidation of receptor-mediated signaling events in time and space will depend on a much deeper understanding of the interactions among receptors and signaling molecules which has recently emerged as an important paradigm in the GPCR field. Beyond receptor oligomerization (Table 1), a number of novel proteins have been found to interact with the α_1_-AR subtypes (Table 2), but for most of these interactions the functional implications are elusive. The vast majority of studies on α_1_-AR subtypes have been performed in recombinant systems. A big challenge in the future will be to explore the functional implications of a variety of interactions in different tissues and physiological conditions. The α_1_-AR subtypes are important regulators of several physiological parameters as highlighted by studies in genetically modified mice (Table 3), and further investigation on this receptor system might have new interesting implications in pharmacology and drug development.
