# Erratum

**DOI:** 10.3109/10799893.2011.597145

**Published:** 2011-07-12

The α1-adrenergic receptors: diversity of signalling networks and regulation, *Journal of Receptors and Signal Transduction*, 2010; 30(6): 410-419.

An error in the print and online versions of this article has been brought to our attention by the authors.

The first line of Table 3 contained an error. The first receptor was incorrectly listed as α1b. It should read: α1a.

The publisher would like to apologize for any inconvenience caused.

